# Supplementary material for: Prior experience of captivity affects behavioural responses to ‘novel’ environments
Source: PeerJ. 2022 Aug 30;10:e13905. doi: 10.7717/peerj.13905 (PMC9438767; doi:10.7717/peerj.13905)
Supplement: Supplemental Information 1 [file peerj-10-13905-s001.docx]

**Supplementary material**

Table S1. An overview of the experiments carried out with great tits (*Parus major*).

Table S2. Multivariate mixed effect models without the 3 birds trapped in Jyväskylä.

Figure S1. Distribution of experienced birds caught over the study period

Video S1. Example of a video of exploration in the room

Video S2. Example of a video of exploration in the cage

Video S3. Example video of the mirror experiment

Table S3. Principal Component loadings

Table S4. Testing the association of the interaction of test-type and prior experience of captivity.

Table S5. Data balance of the discrete variables

Table S6. Results of a multivariate mixed model with ‘prior experience of captivity’ as a binary variable (‘Experience of captivity’).

Figure S2. Number of times caught and total amount of days spent in capitivity

Table S7. The relationship of the number of times a bird was caught and exploration behaviour.

Figure S3. Adjusted repeatability plots without and with controlling for captive experience

Data and R-code belonging to the Manuscript can be found through this link:

https://doi.org/10.5281/zenodo.6865934

Table S1. **An overview of the experiments carried out with great tits (*Parus major*) during prior captivity (N=26)**. Many birds were used in multiple experiments during the same capture event, or were recaptured and tested in a subsequent experiment. The exact nature of the experiments and methods used can be found in the respective references, when the data has been used for publication.

| **Type of experiment** | **Number of birds used** | **Reference** |
| --- | --- | --- |
| Feeding trials using great tits:  1) To test the relative palatability of putatively aposematic mimetic moth species without visual cues.  2) Testing for generalized avoidance between a putative mimetic pair of wood tigermoths (*Arctia* *plantaginis*) | 6 | Rönkä K, Mappes J, Michalis C, Kiviö R, Salokannas J, Rojas B. 2018. Can multiple-model mimicry explain warning signal polymorphism in the wood tiger moth, Arctia plantaginis (Lepidoptera: Erebidae)? Biological *Journal of the Linnean Society*, 124: 237-260. |
| Colour preference tests using palatable and unpalatable moths. | 8 | Rojas B, Rönkä K, Nokelainen O, unpublished data |
| Social avoidance learning using video playback and unpalatable (chloroquine) Tenebrio worms and novel paper prey items. | 7 | Hämäläinen L, Mappes J, Rowland HM, Thorogood, R. 2019. Social information use about novel aposematic prey is not influenced by a predator's previous experience with toxins. *Functional Ecology*, 33: 1982-1992.  Hämäläinen L, Mappes J, Rowland H, Teichmann M, Thorogood, R. 2020. Social learning within and across predator species reduces attacks on novel aposematic prey. *Journal of Animal Ecology*, 89: 1-12. |
| Colour preference tests using palatable and unpalatable (chloroquine) almond flakes | 5 | Teichmann M, Thorogood R, Hämäläinen L. 2020. Seeing red? Colour biases of foraging birds are context dependent. *Animal Cognition* 23: 1007–1018 |
| Test how exposure and orange band length of caterpillars of wood tigermoths (*Arctia* *plantaginis*) affected relative predation risk by predators (great tits). | 2 | Nielsen ME, Mappes J. 2020. Out in the open: behavior’s effect on predation risk and thermoregulation by aposematic caterpillars. *Behavioral Ecology*, 31: 1031-1039 |
| Investigating whether transparency of butterfly wings is associated with decreased detectability by great tits. | 10 | Arias M, Mappes J, Desbois C, Gordon S, McClure M, Elias M, Nokelainen O, Gomez D. 2019. Transparency reduces predator detection in mimetic clearwing butterflies. *Functional Ecology*, 33: 1110-1119. |
| Feeding trial on great tits, scoring disgust behaviours to compare the palatability of *Heliconius numata* to one of its locally monomorphic co-mimics (*Mechanitis polymnia*) and to two other locally monomorphic *Heliconius* species (*H. melpomene* and *H. erato*), using experiments designed to eliminate the confounding effect of visual information. | 5 | Arias M, Mappes J, Théry M, Llaurens V. 2016. Inter-species variation in unpalatability does not explain polymorphism in a mimetic species. *Evolutionary Ecology*, 30: 419-433. |
| Wild caught great tits were used as model predator, and their reaction during feeding was tested towards five animated images on a computer display to test whether the eye-mimicry or the conspicuousness hypothesis better explain eyespot efficacy. | 2 | De Bona S, Valkonen JK, López-Sepulcre A, Mappes J. 2015. Predator mimicry, not conspicuousness, explains the efficacy of butterfly eyespots. *Proceedings of the Royal Society B: Biological Sciences*, 282: 20150202. |
| Test whether moth frequency affects predation (eaten vs. rejection) by great tits and thus affects the survival of three coexisting morphs of the aposematic wood tiger moth *A. plantaginis.* | 1* | Gordon S, Burdfield-Steel E, Kirvesoja J, Mappes J. 2021. Safety in numbers: How color morph frequency affects predation risk in an aposematic moth. *The American Naturalist*, 198: 128-141. |
| Test the effects of frequency on predation by great tits of three coexisting morphs of the aposematic wood tiger moth in semi-natural conditions outdoors | 6 | Nokelainen O, Gordon S. unpublished data |

* The study by Gordon et al. (2021) used the same room as the room we used for exploration in our study. However, the layout of the room (see figure 1 in Gordon et al. 2021) and the methods carried out in this study are different from the exploration test in our study. The bird used by Gordon et al. (2021) was tested more than 5 months before our exploration tests.

Table S2. **Multivariate mixed effect models without the 3 birds trapped in Jyväskylä.** Results from two multivariate mixed effect models on the behaviours of 50 great tits caught around the Konnevesi research station. The fixed effect coefficients (β) and random effect parameters (σ^2^) with their 95% credible intervals (CI). (i) Model 1 included prior experience of captivity as a continuous variable (Days in captivity); (ii) Model 2 included it as a binary variable (experience of captivity (0/1)). Significant effects (credible intervals not crossing 0) are given in bold.

|  | **Exploratory behaviour moves in 2min**  **N= 53** | **Breathing rate breaths s^-1^**  **N= 53** | **Social interaction – PC1 (Close to and pecking at mirror, movements (+))**  **N= 53** |
| --- | --- | --- | --- |
| **Fixed effects** | **β (95% CI)** | **β (95% CI)** | **β (95% CI)** |
| Intercept | **1.86 (0.97 – 2.95)** | **2.37 (2.22 – 2.51)** | -0.43 (-1.36 – 0.30) |
| Bodymass | 0.03 (-0.48 – 0.55) | 0.06 (-0.02 – 0.15) | -0.31 (-0.80 – 0.18) |
| Sex (male) | 0.31 (-0.65 – 1.22) | **-0.28 (-0.45 – -0.12)** | 0.46 (-0.31 – 1.38) |
| Age (old) | -0.06 (-1.02 – 0.94) | -0.05 (-0.23 – 0.13) | 0.65 (-0.38 – 1.61) |
| Catching date | 0.40 (-0.21 – 1.00) | -0.05 (-0.15 – 0.05) | -0.23 (-0.81 – 0.33) |
| Test type (cage) | **-0.93 (-1.75 – -0.18)** | -- | -- |
| Test order | -0.37 (-1.15 – 0.45) | -- | -- |
| (i) Model 1: continuous measure of captivity |  |  |  |
| Days in captivity | **1.44 (0.47 – 2.48)** | -0.09 (-0.25 – 0.09) | 0.37 (-0.55 – 1.38) |
| Days in captivity * Age (old) | **-1.39 (-2.48** – **-0.31)** | 0.04 (-0.15 – 0.22) | -0.37 (-1.38 – 0.70) |
| (ii) Model 2: binary measure of captivity |  |  |  |
| Experience of captivity | 1**.92 (0.35 – 3.37)** | -0.02 (-0.29 – 0.24) | 0.41 (-0.45 – 1.42) |
| Experience of captivity * Age (old) | **-1.94 (-3.91 – -0.01)** | -0.04 (-0.42 – 0.31) | -0.40 (-2.52 – 1.39) |
| **Random effects** | **σ2 (95% CI)** |  |  |
| Bird_ID | 0.40 (<0.01 – 1.38) |  |  |
| Residual | 3.44 ( 2.03 – 5.16) |  |  |
|  | **Link-scale approximation**  **R (95% CI)** |  |  |
| **Repeatability (adjusted)** | 0.10 (< 0.01 – 0. 31)^&^ |  |  |

^&^ because the variance components are constrained to be positive in MCMCglmm models, a lower bound of the credible interval close to zero indicates low confidence in a non-zero proportion of the phenotypic variance in exploratory behaviour being explained by differences between individuals.

Figure S1. **Distribution of birds with experience of captivity (yes/no) caught over the study period**. In total 53 great tits were caught in 2018 over the period: 16^th^ – 31^st^ of March, birds with experience of captivity are in blue, birds without experience of captivity are in orange.

Videos S1, S2 & S3 are 1-minute clips from the videos we analysed for room exploration, cage exploration and social response behaviour.

Video S1. **An example video clip of room exploration.** [*https://doi.org/10.5281/zenodo.5938353*](https://doi.org/10.5281/zenodo.5938353)

Video S2. **An example video clip of cage exploration.** [*https://doi.org/10.5281/zenodo.5938353*](https://doi.org/10.5281/zenodo.5938353)

Video S3. **An example video clip of the social response behaviour towards the mirror.** [*https://doi.org/10.5281/zenodo.5938353*](https://doi.org/10.5281/zenodo.5938353)

Table S3. **Principal Component (1-3) loadings of the variables in the PCA**. The first Principal Component (PC1; explaining 41% of the variation) represented interaction with the mirror: number of pecks on the mirror, relative time spent on the perch closest to the mirror, and increased movement are positively loaded while relative time spent on the perch furthest from the mirror is negatively loaded. Subsequent PC’s (PC2: 24% & PC3: 23%) were dominated by time spent looking at the mirror (PC2) and increased movement (PC3).

| **Variable** | **PC1** | **PC2** | **PC3** |
| --- | --- | --- | --- |
| Difference in time spent of Perch 2 (far and out of view from mirror) | -0.231 | -0.369 | 0.767 |
| Difference in time spent of Perch 5 (near mirror) | 0.653 | -0.031 | 0.033 |
| Difference in number of movements | 0.363 | 0.366 | 0.630 |
| Time spent looking at the mirror | 0.136 | -0.843 | -0.045 |
| Number of pecks at the mirror | 0.608 | -0.137 | -0.110 |

Table S4. **Testing the association of the interaction of test-type and prior experience of captivity using a univariate model.** The fixed effect coefficients (β) and random effect parameters (σ^2^) with their 95% credible intervals (CI) based on two univariate mixed effect models on exploration behaviour in 53 great tits. (i) Model 1 included captivity experience as a continuous variable (Days in captivity), (ii) Model 2 included prior experience of captivity as a binary variable (Experience of captivity (0/1)). Significant effects (credible intervals not crossing 0) are given in bold. In both models we tested whether the interaction of test type * captivity experience was associated with the exploration behaviour.

|  | **Exploratory behaviour moves in 2min**  **N= 53** |
| --- | --- |
| **Fixed effects** | **β (95% CI)** |
| Intercept | 0.86 (-0.10 – 1.86) |
| Bodymass | 0.05 (-0.42 – 0.56) |
| Sex (male) | 0.39 (-0.47 – 1.25) |
| Age (old) | 0.07 (-0.89 – 1.07) |
| Catching date | 0.45 (-0.14 – 1.08) |
| Test type (cage) | **-0.92 (-1.79 – -0.13)** |
| Test order | -0.43 (-1.20 – 0.30) |
| (i) Model 1: length of captivity |  |
| Days in captivity | **1.73 (0.67 – 2.86)** |
| Days in captivity * Age(old) | **-1.50 (-2.56** – **-0.38)** |
| Days in captivity * Test type (cage) | 0.06 (-0.70 – 0.80) |
| (ii) Model 2: binary captivity |  |
| Experience of captivity | **2.26 (0.56 – 4.22)** |
| Experience of captivity * Age(old) | **-2.11 (-4.13 – -0.19)** |
| Experience of captivity * Test type (cage) | -0.25 (-1.78 – 1.36) |
| **Random effects** | **σ2 (95% CI)** |
| Bird_ID | 0.49 (<0.01 – 1.50) |
| Residual | 3.38 ( 1.79 – 4.86) |

Table S5. **The balance of the data according to the different categorical variables for the 53 great tits in our study.**

|  | | Sex | | Age | |
| --- | --- | --- | --- | --- | --- |
|  |  | Female | Male | Young | Old |
| Age | Young | 13 | 19 |  | |
|  | Old | 9 | 12 |  |  |
| Experience with captivity | Yes | 11 | 15 | 11 | 15 |
|  | No | 11 | 16 | 21 | 6 |

Table S6. **Results of a multivariate mixed model with ‘prior experience of captivity’ as a binary variable (‘Experience of captivity’).** The fixed effect coefficients (β) and random effect parameters (σ^2^) with their 95% credible intervals (CI) based on a multivariate mixed effect model on three behaviours in 53 great tits. Significant effects (credible intervals not crossing 0) are given in bold.

|  | **Exploratory behaviour moves in 2min**  **N= 53** | **Breathing rate (breaths s^-1^ )**  **N= 53** | **Social interaction – PC1 (Close to and pecking at mirror(+))**  **N= 53** |
| --- | --- | --- | --- |
| **Fixed effects** | **β (95% CI)** | **β (95% CI)** | **β (95% CI)** |
| Intercept | 0.50 (-0.69 – 1.57) | **2.41 (2.23 – 2.59)** | **-0.79 (-1.73 – -0.01)** |
| Bodymass | 0.04 (-0.43 – 0.55) | **0.09 (0.01 – 0.19)** | -0.34 (-0.79 – 0.13) |
| Sex (male) | 0.32 (-0.58 – 1.25) | **-0.28 (-0.45 – -0.12)** | 0.47 (-0.51 – 1.24) |
| Age (old) | 1.34 (-0.02 – 2.81) | 0.01 (-0.28 – 0.28) | 0.86 (-0.50 – 2.18) |
| Catching date | **-**0.03 (-0.14 – 0.09) | -0.03 (-0.14 – 0.08) | -0.16 (-0.79 – 0.40) |
| Test type (cage) | **-0.90 (-1.78 – -0.16)** | -- | -- |
| Test order | -0.42 (-1.14 – 0.40) | -- | -- |
| Experience of captivity | **2.15 (0.78 – 3.79)** | -0.04 (-0.31 – 0.24) | 0.44 (-0.81 – 1.95) |
| Experience of captivity * Age (old) | **-2.07 (-3.88 – -0.15)** | -0.13 (-0.48 – 0.21) | -0.30 (-2.07 – 1.38) |
| **Random effects** | **σ2 (95% CI)** |  |  |
| Bird_ID | 0.54 (<0.01 – 1.68) |  |  |
| Residual | 3.37 ( 2.05 – 4.88) |  |  |


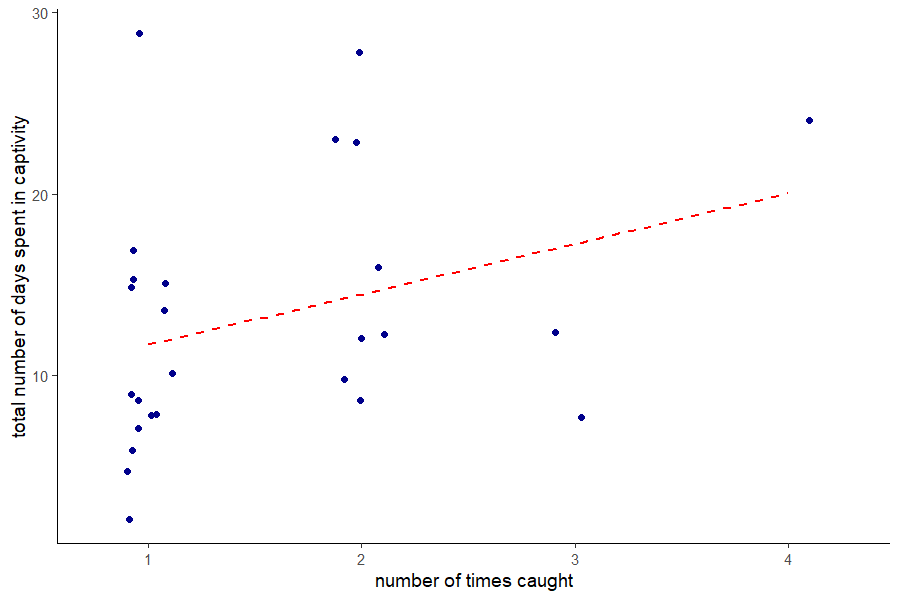
Figure S2**. Relationship between the number of times caught** **and total amount of days spent in capitivity**. We here show a plot of the 26 caught great tits with their total amount of days spent in captivity relative to the number of times they were caught prior to our capture. We show here that there is no significant relationship in our data between the number of times a bird was caught and the length of captivity that birds experienced. The non-significant relationship is indicated by the red dashed line, significance was tested using a negative binomial glm of ‘days in captivity’ as a function of ‘number of times caught’: coeff. = 0.19, std.error = 0.12, p = 0.10. Data points are jittered for visual purposes.

Table S7. **The relationship between the number of times a bird was caught and exploration behaviour.** Associations from a linear mixed model where we test whether there is a relationship between the number of times a bird was caught and the exploration behaviour, we find no significant relationship. We used the same fixed effect structure as in our main models (see *Table 2*) and for the random effect we used bird id. Random effect statistics are not given, as we are interested mainly in the effect of number of times a bird was caught, N = 53 birds.

|  | **Exploratory behaviour moves in 2min**  **N= 53** | | |
| --- | --- | --- | --- |
| **Fixed effects** | **β (std. error)** | **z value** | **P(z)** |
| Intercept | 2.35 (0.40) | 5.93 | < 0.001 |
| Bodymass | 0.02 (0.18) | 0.10 | 0.921 |
| Sex (male) | 0.21 (0.32) | 0.65 | 0.517 |
| Age (old) | -0.06 (0.35) | -0.17 | 0.869 |
| Times caught | 0.57 (0.36) | 1.55 | 0.121 |
| Catching date | 0.31 (0.23) | 0.65 | 0.167 |
| Test type (cage) | -0.11 (0.33) | 0.24 | 0.814 |
| Test order | -0.30 (0.40) | -0.94 | 0.348 |
| Days in captivity * Age (old) | -0.48 (0.40) | -1.19 | 0.233 |


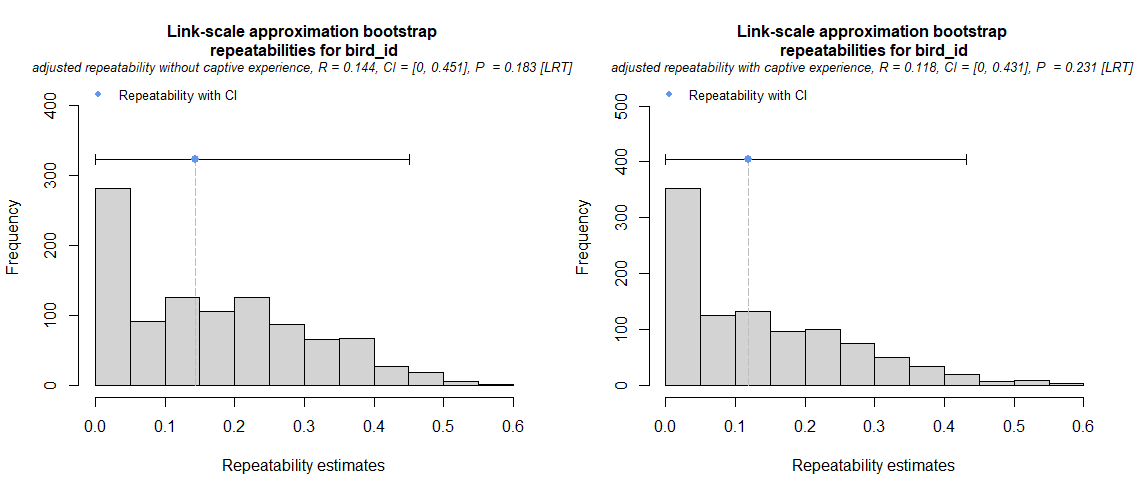


Figure S3. **Adjusted repeatability plots without and with controlling for captive experience (continuous).** Plots of adjusted repeatability of exploratory behaviour of 53 great tits tested in an exploration room and cage. Left plot showing the adjusted repeatability, without controlling for captive experience (continuous), model 1. Right plot showing adjusted repeatability with captive experience in the model (2). Analyses done using r-package ‘rptr’ (Stoffel et al. 2017). Approximation for the repeatability and CI was done using 1000 bootstraps. A Poisson distributed generalized linear mixed-effects model was used:

model 1: exploration_2min ~ test (cage/room) + test.order + (1|Bird_ID)

model 2: exploration_2min ~ test (cage/room) + test.order + days_captivity + (1|Bird_ID)

Reference:

- Stoffel MA, Nakagawa S, Schielzeth H. 2017. rptR: repeatability estimation and variance decomposition by generalized linear mixed-effects models. Methods in Ecology and Evolution, 8: 1639-1644. doi:10.1111/2041-210X.12797
